# Sensing Features of Long Period Gratings in Hollow Core Fibers

**DOI:** 10.3390/s150408009

**Published:** 2015-04-03

**Authors:** Agostino Iadicicco, Stefania Campopiano

**Affiliations:** Engineering Department, University of Naples “Parthenope”, Centro Direzionale Isola C4, 80143 Napoli, Italy

**Keywords:** long period grating, hollow core fiber, pressure sensing, strain sensing, electric arc discharge

## Abstract

We report on the investigation of the sensing features of the Long-Period fiber Gratings (LPGs) fabricated in hollow core photonic crystal fibers (HC-PCFs) by the pressure assisted Electric Arc Discharge (EAD) technique. In particular, the characterization of the LPG in terms of shift in resonant wavelengths and changes in attenuation band depth to the environmental parameters: strain, temperature, curvature, refractive index and pressure is presented. The achieved results show that LPGs in HC-PCFs represent a novel high performance sensing platform for measurements of different physical parameters including strain, temperature and, especially, for measurements of environmental pressure. The pressure sensitivity enhancement is about four times greater if we compare LPGs in HC and standard fibers. Moreover, differently from LPGs in standard fibers, these LPGs realized in innovative fibers, *i.e.*, the HC-PCFs, are not sensitive to surrounding refractive index.

## 1. Introduction

The need for real time, *in situ* monitoring of physical, chemical and biological parameters is increasing in various commercial and defense fields. Sensor technology has become a basic enabling technology in many instances as several different applications push their adoption from security systems to environmental monitoring, from structural health monitoring to medical diagnostics [1,2,3].

In particular, optical fiber sensors play an important rule due to their unique characteristics of high performances and reliable sensors [4]. Their properties arise from their compact size, all-optical readout, inmunity to electromagnetic interference, remote sensing ability, and more.

Among all the optical fiber sensors, a lot of attention has been paid to Long-Period Gratings (LPGs) which find broad use in many applications [5]. Indeed, LPGs can have properties designed to fulfill specific parameters of interest with sensitivities higher than possible with classical fiber grating sensors. 

LPGs are formed by longitudinal periodic refractive index and/or structural modulation along an optical fiber that have periods typically in the range 100 μm to 1 mm, and thus they promote coupling between the propagating core mode and co-propagating higher order modes [5]. These modes decay rapidly as they propagate along the fiber axis resulting in the transmission spectrum containing a series of attenuation bands centered at discrete wavelengths, corresponding to the coupling with a different cladding mode. The form and the center wavelengths of the attenuation bands, are sensitive to the period of the LPG, to the length of the LPG (typically of the order of 30 mm) and to the local environment making them attractive for applications in sensing strain, temperature, bend radius and external index of refraction [5].

Typically LPGs are fabricated as standard single mode fibers (SMFs) by different techniques including UV laser exposure, electric arc discharge, chemical etching, and irradiation by femtosecond laser pulses and CO_2_ lasers. Efforts have also been done in order to enhance the performance of LPGs in single mode fibers in terms of tuning capability and/or sensitivity, like the deposition of thin high RI (HRI) layer onto the cladding over the grating region [6].

On the other hand, it is worth noting that innovative fibers like hollow core photonic crystal fibers (HC-PCFs), also named photonic bandgap fibers (PBFs), capable to offer new perspective in sensing and telecommunications applications have attracted the attention of several research groups and scientists since the last years [7,8,9]. The emergence of HC-PCFs has resulted in LPGs inscribed in them with novel properties. LPGs in HC-PCFs provide new promising platforms for developing novel sensing devices by combining the unique characteristics of hollow core fibers with the properties of LPGs.

The first demonstration of LPG in HC-PCF can be traced back to 2008 [10,11]: the hard challenging manufacturing of LPG in HC-PCF is achieved by using a pulsed CO_2_ laser that modifies the micro-structures along the fiber. The laser pulses hit repeatedly one side of the HC-PCFs inducing localized changes in shape, size, and some air holes collapse in the cladding. However the final device is very fragile and strongly polarization dependent.

More recently, in order to overcome the drawbacks of the CO_2_-based fabrication technique, the authors of the present work have demonstrated that LPGs in HC-PCFs can be fabricated by a pressure assisted Electric Arc Discharge (EAD) technique [12,13,14]. For the first time the EAD procedure was combined with pressure inside the fiber hole permitting to avoid the cladding lattice collapsing [15]. It is worth noting that, currently, it seems the unique solution to realize LPGs without any polarization dependence. In this paper, authors present a thorough analysis of the sensing features of the LPGs fabricated in HC-PCFs by pressure assisted EAD technique. The achieved results show that LPGs in HC-PCFs represent a novel high performance sensing platform for measurement of different physical parameters including strain, temperature and especially for measurements of environmental pressure. Additionally it is worth noting that, differently from LPGs in standard fibers, these novel gratings are not sensitive to surrounding refractive index, whereas LPGs in standard fibers received the most of their popularity in (bio)chemical application field thank to this sensing feature itself. However, although not yet investigated, we believe that this limitation can be overcome by taking advantage by the air core characteristic of the hosting hollow core fiber, as proposed by recent papers [9].

## 2. Long Period Gratings in Hollow Core Fiber

The LPGs were manufactured in a commercial HC fiber (HC-1550-02, NKT Photonics, Birkerød, Denmark) with a step by step approach based on electric arc-discharge (EAD) technique properly modified to meet the fiber requirements [12]. The use of EAD procedure combined with a proper fiber axial tension is widely promoted as non-UV method to realize LPGs in single mode fibers (SMFs) [16] and successively applied to PCFs with solid cores [17]. The main effect consists in a periodic perturbation (called tapering) of the transversal size of the core and cladding regions along the fiber axis [16]. Moreover, the silica refractive index changes due to stress relaxation induced by local hot spots also contribute to the grating formation.

The capillaries forming the holey fiber structure seem too thin for the use of the standard EAD procedure to realize LPGs. Indeed, typically, the standard EAD treatment on HC-PCFs leads to the localized collapse of the innermost ring of the cladding air holes with dramatic consequences for the HC fiber propagation features [18].

Recently we demonstrated that drawbacks, like the collapsing of the capillaries, can be overcome by combining a properly weak EAD step with a slight static pressure inside the HC fiber holes [12,13,14,15]. Despite the fabrication of LPG in SMFs, in the current procedure there is no axial tension along the fiber. Rather the hollow core fiber is kept well aligned in between splicer electrodes and left free from any mechanical stress by home-made holders. Then the arc discharge procedure is provided when a static pressure is forced inside the fiber holes with fusion current and arc duration significantly lower than standard values used to realize LPGs in SMFs. The effect is a slight localized modification of the size and shape of core and cladding holey structure permitting an effective refractive index modulation and avoiding any hole collapsing. Since a LPG consists in a periodic perturbation, the procedure is then periodical repeated along the fiber length with period of Λ by means of a microstepper with resolution of 1 μm.

**Figure 1:**
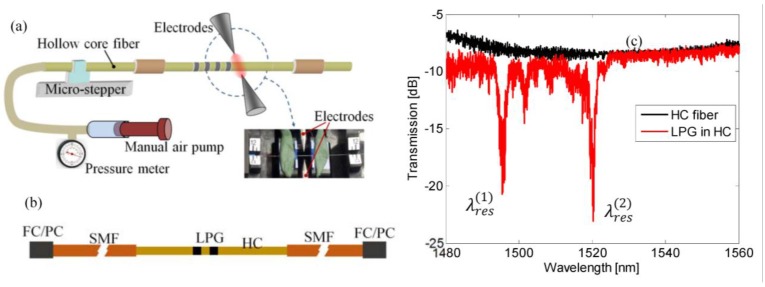
(**a**) Schematic diagram of LPG fabrication experimental setup; (**b**) Schematic view of HC fiber with grating spliced with conventional single mode fibers with FC/PC connectors; (**c**) Transmitted spectra of HC fiber spliced with conventional SMFs with FC/PC connectors with and without LPG.

In Figure 1, a schematic diagram of the experimental setup is shown [12]. The arc discharges have been carried out by a commercial fusion splicer unit (Type-39, Sumitomo Electric Lightwave Corp, Research Triangle Park, NC, USA) with fusion current and arc duration manually fixed to three steps (properly scaled, where the third step is approximately 13 mA) and 400 ms, respectively. Besides, to force a static pressure inside the fiber holes, one end of the hollow-core fiber was connected to a small air pump while the second end was plugged by EAD treatment. During the EAD procedure the static pressure inside core and cladding holes was kept constant at 126 kPa ± 0.1 kPa and monitored via a pressure meter.

Once the grating is manufactured, the HC fiber ends (with gratings in the middle) were properly cleaved and spliced to conventional single mode fibers with FC/PC connectors, as schematically plotted in Figure 1b, by a manually optimized procedure as in [19]. Transmitted spectra of the HC-LPGs were measured by means of a broadband source (SLED around 1550 nm) and an optical spectrum analyzer (Yokogawa AQ6370B, Tokyo, Japan) with 20 pm resolution.

Figure 1c compares the spectrum of a pristine HC fiber (spliced in between two SMFs) and the spectrum of a LPG with pitch of Λ = 400 μm and length of 25 periods, L = 25 Λ. The grating transmitted spectrum clearly exhibits the presence of two attenuation bands due to resonant coupling of the fundamental core mode to higher order modes at $\lambda_{res}^{\left(1\right)}=1495.4\;\mathrm{nm}$ and $\lambda_{res}^{\left(2\right)}=1520.3\;\mathrm{nm}$ with attenuation depth of 9.4 dB and 11.9 dB and bandwidth of 1.4 nm and 1.5 nm, respectively. The resonant wavelengths depend on the grating features by the well-known coupled-mode theory formalism, which is well suited for LPGs based on weak and uniform index modulations:

(1) $$N\cdot \lambda_{res}^{\left(i\right)}=\left(n_{F}-n_{H}^{\left(i\right)}\right)\cdot \Lambda$$

where N, $n_{F}$, $n_{H}^{\left(i\right)}$ and Λ are, respectively, the grating order, the effective indexes of the fundamental mode and i-th higher order mode, and the grating period. It is worth noting that at current state of our study the order and shape of the modes involved in the resonant coupling mechanism are not completely known. Numerical analysis based on finite element method are currently in progress to fully meet this requirement.

Moreover, it is also possible to observe background oscillations in the two spectra, with and without LPG, attributable to different effects: (i) Fabry-Perot effect due to HC-SMF splicing (ii) higher order modes (HOM) weakly excited in the HC fiber [12].

**Figure 2:**
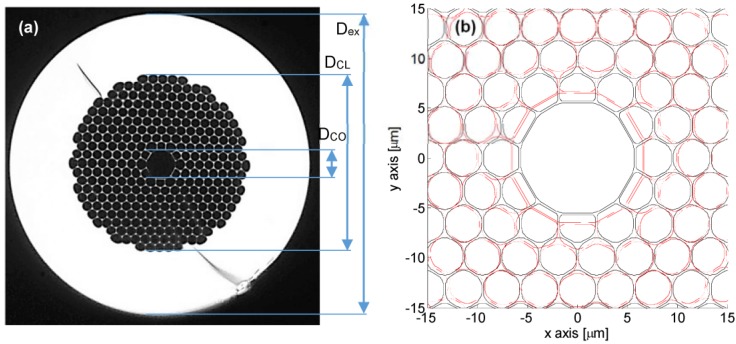
(**a**) Microscope image of the pristine fiber cross section; (**b**) Schematic view/comparison of the cross section of the pristine and perturbed fibers (achieved via Matlab): Solid black line is the pristine fiber, and dashed red line is the perturbed fiber.

In order to understand the effect of the pressure assisted EAD discharge procedure, a morphological characterization of the cross sections of fibers before and after EAD procedure by means of microscope image analysis is discussed in [15]. Briefly, Figure 2a shows a cross view of the pristine fiber where the external diameter is about D_ex_ = 120 μm. The microstructured cladding of the fiber (with diameter of approximately D_CL_ = 70 μm) is formed by hexagonal holes with rounded corners arranged in a triangular lattice (with pitch of 3.8 ± 0.1 μm—From datasheet). The core is formed by omission of seven holes and it is often represented by a circular-like hole with diameter of D_CO_ = 11 ± 0.5 μm. However, the exact core/cladding interface exhibits a dodecagonal shape with rounded corners due to the connection between core region and the 12 holes of the first cladding ring. To fit the core shape the holes of the first cladding ring are slightly different from the remnant of the cladding region: the first ring indeed includes six non-regular hexagonal holes and six non-regular pentagonal holes alternatively arranged.

The microscope image analysis as in [15] reveals that the main effect consists in the core up-tapering, that is an enlargement of the core size (diameter) passing from D_CO_ = 11.0 ± 0.5 μm to D_CO_ = 13.0 ± 0.5 μm. Additionally, it is possible to retrieve that the external diameter of the fiber is reduced from D_ex_ = 120 ± 0.5 μm to D_ex_ = 117 ± 0.5 μm as well as the inner diameter of the external solid silica region from approximately D_CL_ = 70.0 ± 0.5 μm to D_CL_ = 65.0 ± 0.5 μm. Figure 2b shows a comparison of the cross section (around the core region) of the pristine and perturbed fibers achieved via Matlab. As evident the EAD procedure induces significant changes in shape and size of the air-hole in the cladding region. In particular it seems that the external rings are decreased in diameter without significant reshape. This behavior changes for inner rings. The innermost rings in-fact seem to change from circularly-hexagonal to elliptically-hexagonal where the minor axis is along the radial direction and the major axis is along the angular direction.

## 3. Sensitivities and Results

This section presents and discusses the sensitivity characteristics of resonant bands of LPG in HC fiber to environmental parameters changes. LPGs exhibit sensitivities to a number of factors and the corresponding resonant wavelengths shifts can be represented by:

(2) $${\delta \lambda }_{res}^{\left(i\right)}=S_{T}^{\left(i\right)}\delta T+\;S_{\varepsilon }^{\left(i\right)}\delta \epsilon +\;S_{n_{out}}^{\left(i\right)}\delta n_{out}+\;S_{C}^{\left(i\right)}\delta C\;+\;S_{P}^{\left(i\right)}\delta P+\;S_{\tau }^{\left(i\right)}\delta \tau$$

where *T*, ε, *n_out_*, *C*, *P* and *τ* stand for the temperature, strain, outside refractive index, curvature, static pressure, and torsion, respectively. 

The sensitivities $S_{T}^{\left(i\right)}$,$\;S_{\varepsilon }^{\left(i\right)}$, $S_{n_{out}}^{\left(i\right)}$, $S_{C}^{\left(i\right)}$, $S_{P}^{\left(i\right)}$ and $S_{\tau }^{\left(i\right)}$ of the i-th resonant wavelength $\lambda_{res}^{\left(i\right)}$ to T, ε, *n_out_*, *C*, *P* and τ, respectively, can be expressed as $S_{\xi }^{\left(i\right)}={\delta \lambda }_{res}^{\left(i\right)}/\delta \xi$ where ξ is T, ε, n_out_, C, P or τ. By using the Equation (1), the sensitivities can be expressed as:

(3) $$S_{\xi }^{\left(i\right)}=\frac{1}{N}\left[\Lambda \frac{\delta (∆n_{res}^{\left(i\right)})}{\delta \xi }+∆n_{res}^{\left(i\right)}\frac{\delta \;\Lambda }{\delta \xi }\right]$$

where $∆n_{res}^{\left(i\right)}=\left(n_{F}-n_{H}^{\left(i\right)}\right)$. The first term of the right side of the Equation (3) takes into consideration the dependence of the field distribution of the fiber modes involved into the coupling mechanism to the changing of the environmental factor whereas the second terms relates the environmental stimulus to the grating period.

In case of HC fibers, which are made by single material and air core, some terms are null or can be ignored. For instance, since all modes involved in the coupling mechanism are guided in the air core, we believe that the dependence of the effective refractive index difference on the curvature and torsion are low. Additionally, it is reasonable to believe that both terms, $\frac{\delta (∆n_{res}^{\left(i\right)})}{\delta n_{out}}$ and $\frac{\delta \;\Lambda }{\delta n_{out}}$, are null as consequence of changes of the outer refractive index and thus $S_{n_{out}}^{\left(i\right)}=0$.

In the following we report on the experimental characterization of the sensing features of LPG in HC fiber with 25 perturbations and pitch of Λ = 400 µm (see Figure 1). In particular sensitivities of both resonance bands to local temperature, strain and pressure have been take into account [12,13,14,15]. Besides, also the hardly changes of the spectral response due to curvature and refractive index is presented and discussed. Additionally, to avoid torsion we keep the LPG straight.

Starting with the refractive index sensitivity characterization, the LPGs written in the HC fiber are immersed into liquids with different refractive indexes (aqueous glycerin solutions of different concentration) ranging from 1.33 to 1.45. The resonant wavelength and peak transmission attenuation hardly change, accordingly with the theoretical analysis. Resonant wavelengths random change within wavelength resolution. Thus, we can confirm that LPG in HC fiber are insensitive to surrounding refractive index (SRI) since the power coupling mechanism involves well confined core modes, whereas the LPGs in the conventional SMFs are very sensitive to SRI, especially when the index is about 1.45 [16,20,21,22].

**Figure 3:**
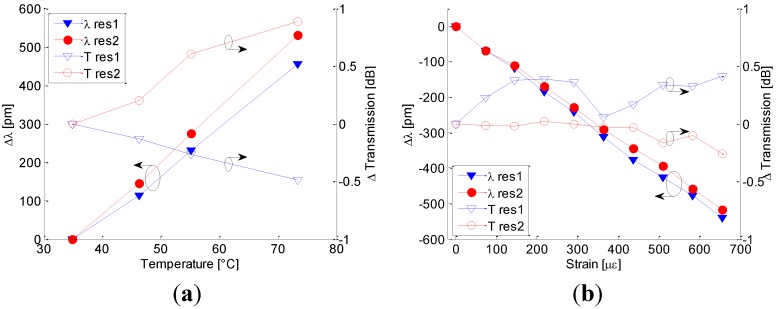
(**a**) Resonant wavelength shifts and peak transmission changes *versus* temperature. (**b**) Resonant wavelength shifts and peak transmission changes *versus* strain.

To examine the temperature sensitivity of LPGs, the ambient temperature of the devices was varied by using a temperature chamber whose temperature can be controlled within the range 30 °C–80 °C and a commercial FBG-based temperature sensor was used as reference. Figure 3a plots the resonant wavelength shifts and peak transmission changes of $\lambda_{res}^{\left(1\right)}$ and $\lambda_{res}^{\left(2\right)}$: in this figure (as well as in the following figures) λres1 and λres2 refer to wavelength shift and Tres1 and Tres2 refer to peak transmission changes of left and right bands. According to the general LPG behavior, attenuation bands exhibit red shift with temperature increasing. Here, sensitivities of $S_{T}^{\left(1\right)}=$ 11.9 pm/°C and $S_{T}^{\left(2\right)}=13.8\;$pm/°C are measured by means of linear fitting with coefficient of determination of R^2^ = 0.9972 and R^2^ = 0.9994 for the first and second attenuation bands, respectively. Because it is expected that all modes involved in the optical power coupling mechanism of the LPGs in HC (fundamental and high order core modes) are guided in the air core [11], it is reasonable to believe that the dependence of the temperature sensitivity on the thermo-optic effect is trivial. On contrary we believe that the main effect of the temperature sensitivity is due to the silica thermal-expansion. The last effect can act on both terms of the right side in the Equation (3): (i) Thermal expansion along fiber axis induces a monotonic change of the period Λ with temperature; (ii) Fiber structure thermal expansion in transversal plan modifies the field distribution and thus the effective refractive indices difference of different core modes.

Besides, for both $\lambda_{res}^{\left(1\right)}$ and $\lambda_{res}^{\left(2\right)}$ negligible changes in peak transmission can be measured even if they monotonically move in opposite direction: the depth of $\lambda_{res}^{\left(1\right)}$ increases of 0.5 dB and the depth of $\lambda_{res}^{\left(2\right)}$ decreases of almost 1 dB when temperature changes from 30 °C to 80 °C. Compared with the LPG in HC fiber fabricated by focused CO_2_ laser (proposed by Wang *et al.* [10]), it exhibits higher sensitivity *versus* thermal changes whereas it is lower than UV or CO_2_ based LPGs in SMFs [16,20,21,23].

The strain sensitivity of LPG in HC fibers was investigated by using a commercial FBG as reference and plotted in Figure 3b. With the increase of applied tensile strain, the resonant wavelengths of our LPGs shift linearly toward shorter wavelength with strain sensitivities of $S_{\varepsilon }^{\left(1\right)}=-0.824$ pm/µε and $S_{\varepsilon }^{\left(2\right)}=-0.781\;$ pm/µε for $\lambda_{res}^{\left(1\right)}$ and $\lambda_{res}^{\left(2\right)}$ with coefficient of determination of R^2^ = 0.9988 and R^2^ = 0.9991, respectively. Similarly to the temperature sensitivity analysis, here it is reasonable that the strain-optic effect is trivial whereas the $S_{\varepsilon }^{\left(1\right)}$ and $S_{\varepsilon }^{\left(2\right)}$ strain sensitivity values can be attributed to both terms of the Equation (3): the first is related to the strain-effect on the fiber transversal size acting on the effective refractive indices and the second one presents a relative period change similar to the strain value.

Additionally, the peak transmission attenuations change within ± 0.5 dB. These sensitivity values are very similar to the sensitivity of LPG in HC fiber realized via CO_2_ laser (−0.830 pm/με [10]). Besides they are slightly higher than the strain sensitivity of UV-based LPG in standard dispersion shifted fiber (about −0.727 pm/με [21]) and about two times the sensitivity of LPG in SMF written by CO_2_ laser pulses (about −0.45 pm/με [23]). Additionally, Bhatia in [20] demonstrated than higher sensitivity can be achieved by changing the attenuation band order reaching sensitivity of −1.94 pm/με and Bock *et al.* [17] demonstrated sensitivity of −2.76 pm/με concerning EAD based LPG written in photonic crystal fibers.

**Figure 4:**
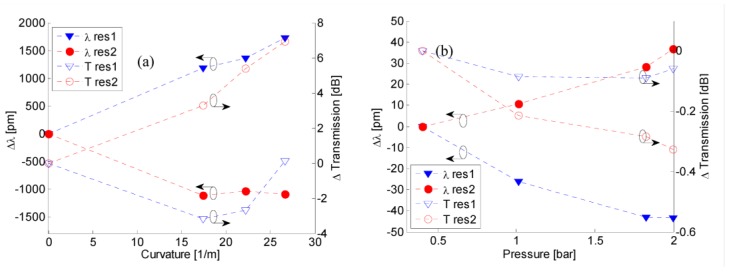
(**a**) Resonant wavelength shifts and peak transmission changes *versus* curvature; (**b**) Resonant wavelength shifts and peak transmission changes *versus* pressure.

Moreover, to investigate the sensitivities to local curvature, transmitted spectra were acquired when the LPG was curved by means of several holders with diameter ranging from 2 × R = 11.5 cm to 2 × R = 7.5 cm. Figure 4a plots the resonant wavelengths shifts and peaks transmission changes of $\lambda_{res}^{\left(1\right)}$ and $\lambda_{res}^{\left(2\right)}$ *versus* local curvature C = 1/R. It is clearly observable that resonant wavelengths and band depths of first and second attenuation bands move in opposite direction when the curvature state changes and thus it is reasonable to believe that $\frac{\delta \;\Lambda }{\delta C}\ll \;\frac{\delta (∆n_{res}^{\left(i\right)})}{\delta C}$. Average sensitivities of $S_{C}^{\left(1\right)}=+64\;\mathrm{pm}\cdot m$ and $S_{C}^{\left(2\right)}=-44\;\mathrm{pm}\cdot m$ are measured for the first and second attenuation bands, respectively. 

The depth of the left attenuation band increases with curvature changing from zero to C = 17.3/m while further increases of the curvature force a decreasing of the band depth close to the pristine value. On contrary the depth of the right band significantly decreases with monotonic behavior when curvature changes from zero to C = 26.7/m. It is worth noting that the above sensitivity values are higher than the ones related to the LPG realized via CO_2_ laser [10]. However, in both cases the sensitivities values are significantly lower than the bending sensitivity of the UV-based LPGs in standard fiber [24,25]. We believe that the significant decreasing in the bending sensitivity of LPGs in HC as compared with the LPGs in SMF is due to field distribution of the modes involved into the power coupling mechanism [26]. In the LPG written in hollow core fiber the optical power coupling is between fundamental core mode and high order core mode, whereas the operation principle of SMF-based grating is based on the core-to-cladding mode coupling. During the fiber bending it is expected that the core lies on the neutral axis of the fiber and its mean axial length hardly change while the fiber bending can induce a complete reshape and/or splitting of the cladding modes [26].

Finally we report on experimental results aimed to test the pressure sensitivities of the LPG in HC fiber. Due to hollow core characteristic and cladding lattice structure it is expected that changes in the surrounding pressure act significantly on the effective refractive indices of the fiber modes and thus on the LPG resonant wavelengths. The selected grating was enclosed in an air pressure chamber equipped with a manual pressure pump and a pressure meter with resolution of 0.05 bar. The pressure was changed from 0.4 to almost 2 bar and the shift of the resonant wavelengths and peak transmission changes of both attenuation bands were plotted in Figure 4b. Like the effect of the local fiber curvature, the resonant wavelengths move in opposite direction when the external static pressure increases: left and right attenuation bands exhibit blue and red shift respectively with not-perfectly linear behavior. However, the linear fitting, shows a pressure sensitivity of $S_{P}^{\left(1\right)}=-26.9\;\mathrm{pm}/\mathrm{bar}$ and $S_{P}^{\left(2\right)}=22.2\;\mathrm{pm}/\mathrm{bar}$ with coefficient of determination of R^2^ = 0.9507 and R^2^ = 0.9834 for $\lambda_{res}^{\left(1\right)}$ and $\lambda_{res}^{\left(2\right)}$, respectively. Concerning to the Equation (3) and taking into consideration the opposite direction of the left and right bands wavelength shift, we believe that the first term (in Equation (3)) should exhibit stronger effect that the second one. The last term in fact exhibit the same sign for both bands.

Moreover negligible changes in the attenuation band depths can be measured. Resonant wavelength shifts are found to be higher than pressure sensitivities of LPGs in single mode fiber and solid core PCF proposed: We have more than double the sensitivity to pressure afforded by a tapered LPGs fabricated in solid core photonic crystal fiber (IG-PCF) proposed by Bock *et al.* [17] and compared to a tapered LPG fabricated in a standard fiber (SMF-28), the sensitivity enhancement is about four times greater [27]. Although a complete understanding has not yet been attained, basic considerations of the experimental data provide sensitivity values typically observed in complex multilayered structures. The probable reason for this is the high air-filling fraction presented in the HC-PCF. Form these preliminary results the development of LPG in HC fiber displays significant potential as a highly sensitive and cost-effective pressure sensor.

**Table 1:** Comparison of sensitivities.

*Grating*	*Ref*	$S_{T}$ [pm/°C]	$S_{\varepsilon }$ [pm/µɛ]	$S_{n_{out}}$ [nm/RIU]	$S_{C}$ [pm·m]	$S_{P}$ [pm/bar]
$\lambda_{res}^{\left(1\right)}$ of this work		+11.9	−0.824	≈ 0	+64	−26.9
$\lambda_{res}^{\left(2\right)}$ of this work		+13.8	−0.781	≈ 0	−44	+22.2
CO_2_ based LPG in HC fiber	[10]	≈+2.9	−0.830	≈0	≈1.2	-
EAD LPG in solid-core PCF	[17]	≈+0.35	−2.76	-	-	+11.2
UV LPG in NRL fiber	[24]	+54.0	−0.15	-	>10^3^	-
UV LPG in Corning SMF-28	[25]	-	-	-	5.2 × 10^3^	-
UV LPG in dispersion shifted fiber	[21]	+62.0	−0.727	−645 (1.40–1.45)	-	-
CO_2_ based LPG in SMF-28	[23]	≈+58.0	≈−0.45	-	−7 × 10^3^	-
EAD LPG in SMF-28	[27]	+49.5	−0.6	-	-	+5.1

A clear comparison of the sensitivities above mentioned is reported in Table 1. Here LPG in different kind of optical fiber and several fabrication approaches are compared. It is worth highlighting that in most of the selected papers the attention is focused on physical parameters sensing and thus the surrounding refractive index sensitivity is not investigated. However the SRI sensitivity remains one of the most appealing feature of the LPG in light of its freedom degrees in the design of the desired sensitivity and refractive index range [6,16].

## 4. Conclusions

This paper presented and discussed the sensing features of recently fabricated long period grating in hollow core fibers. As reported in previous works, the LPG were fabricated in hollow core fibers by means of a modified EAD procedure assisted by fiber pressurization allowing the achievement of a sufficient effective refractive index modulation for resonant mechanism and at the same time preserving the bandgap features of the host fibers.

The characterization of the LPG sensitivity to environmental parameters such as strain, temperature, curvature, refractive index and pressure has been presented here. These results highlight that the temperature sensitivity is significantly lower than LPGs in SMFs whereas strain sensitivity is higher than LPGs. Additionally a negligible sensitivities were measured *versus* the curvature changes and in particular *versus* the surrounding refractive index changes. Finally high sensitivities *versus* environmental static pressure is measured displaying significant potential to design novel highly sensitive and cost-effective pressure sensors. We would like to point out that the temperature and strain sensitivities of the left and right resonant wavelengths are similar (shift toward one direction), while the resonant wavelengths for $\lambda_{res}^{\left(1\right)}$ and $\lambda_{res}^{\left(2\right)}$ exhibit blue and red shift *versus* external pressure, respectively. Currently FEM analysis aimed to study the dependence of the core modes involved in the coupling mechanism on environmental parameters are in progress.

Finally, we believe that the assessment of such technology permits one to improve the sensing performance of the hollow core fibers. Unique features of the hollow core fibers in terms of light propagation within the air core and large internal surface have attracted the attention of several research groups for application in sensing and communication fields. We believe that it is desirable to combine such as features with the grating devices in light of their spectral characteristics and wavelength encoded measurements.
